# About Pyrimidine 5’-Nucleotidase Deficiency

**DOI:** 10.4274/Tjh.2013.0050

**Published:** 2013-06-05

**Authors:** Şinasi Özsoylu

**Affiliations:** 1 Fatih University, School of Medicine, Department of Hematology, Ankara, Turkey

## TO THE EDITOR

Agapidou et al.’s case of pyrimidine 5’-nucleotidase deficiency and heterozygous α-thalassemia was an interesting co-existence [1]. Did the authors have the chance to study the offspring for these hereditary conditions from birth?

We also reported a family with severe hereditary pyrimidine 5’-nucleotidase deficiency; in addition, 3 other families were also mentioned [2]. Since then 5 other patients also received this diagnosis. Therefore, I believe that this deficiency is not very rare in this area.

## REPLY

**Dear Mr Şinasi ÖZSOYLU**

I would like to thank you for your interest in our work. Concerning our manuscript entitled ‘”Co-Existence of Hereditary Pyrimidine 5-Nucleotidase Deficiency and Heterozygous α-Thalassemia” published in TJH, the specific patient was diagnosed during her early childhood in NIMITS hospital, Athens by Prof Tegos. She came to our center, in General Hospital Hippokratio Thessaloniki, Prevention Unit for her prenatal control and at that time we discovered that she was also heterozygous in a-thalassemia. We consider it an interesting case report because even though pyrmidine 5’-nucleotidase deficiency is not very rare in the area, it is quite difficult to diagnose it due to lack of specific centers dealing with such enzymic deficiencies and furthermore we describe a co-existence of pyrimidine 5’-nucleotidase deficiency and heterozygous a-thalasemia which is rare according to international literature. We are looking forward to further reviews.

**Alexandra Agapidou**
